# Supplementary figures and images for: How do e-commerce platforms and retailers implement discount pricing policies under consumers are strategic?
Source: PLoS One. 2024 May 10;19(5):e0296654. doi: 10.1371/journal.pone.0296654 (PMC11086857; doi:10.1371/journal.pone.0296654)

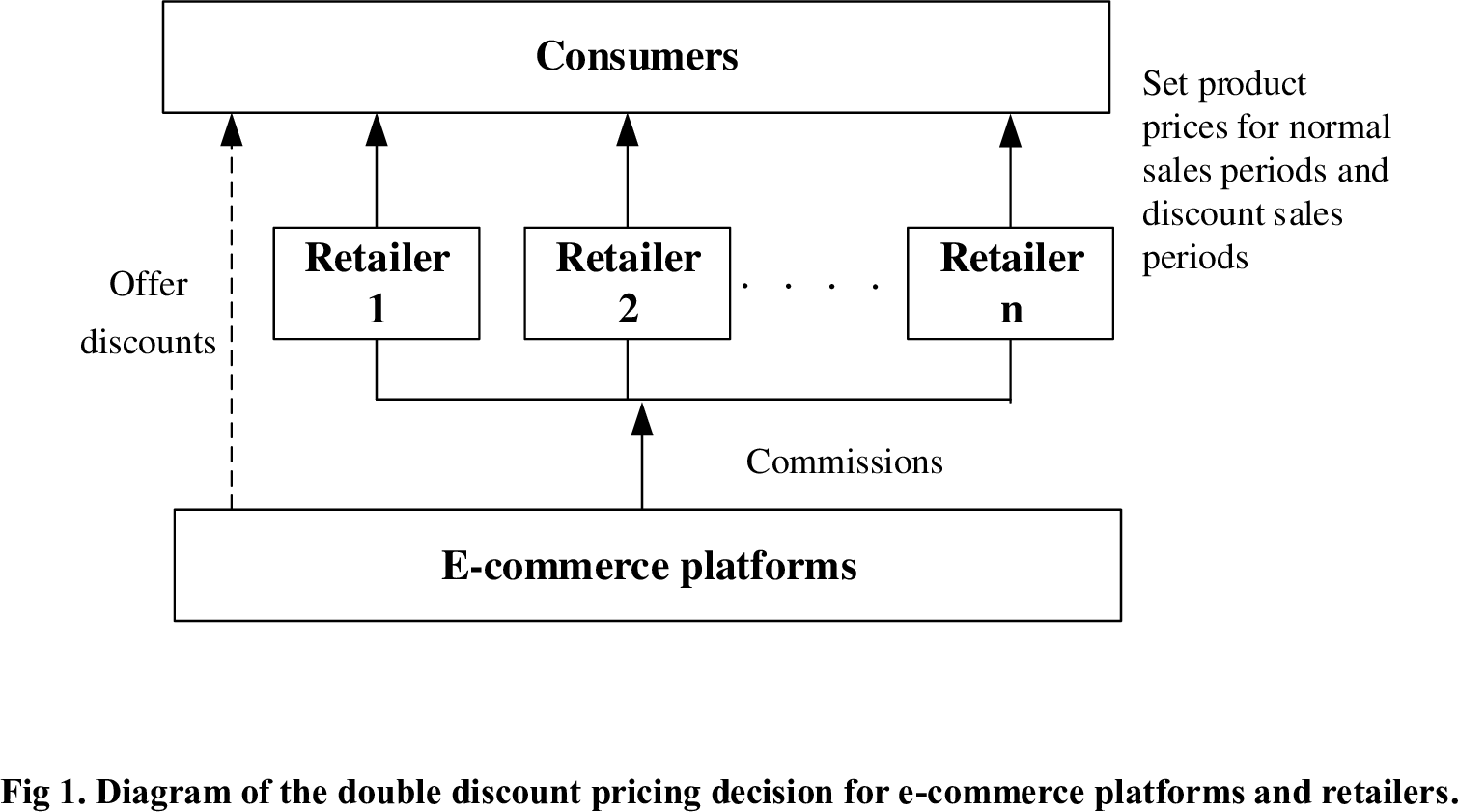

Supplement: S1 Fig — (TIF) [file pone.0296654.s001.tif]

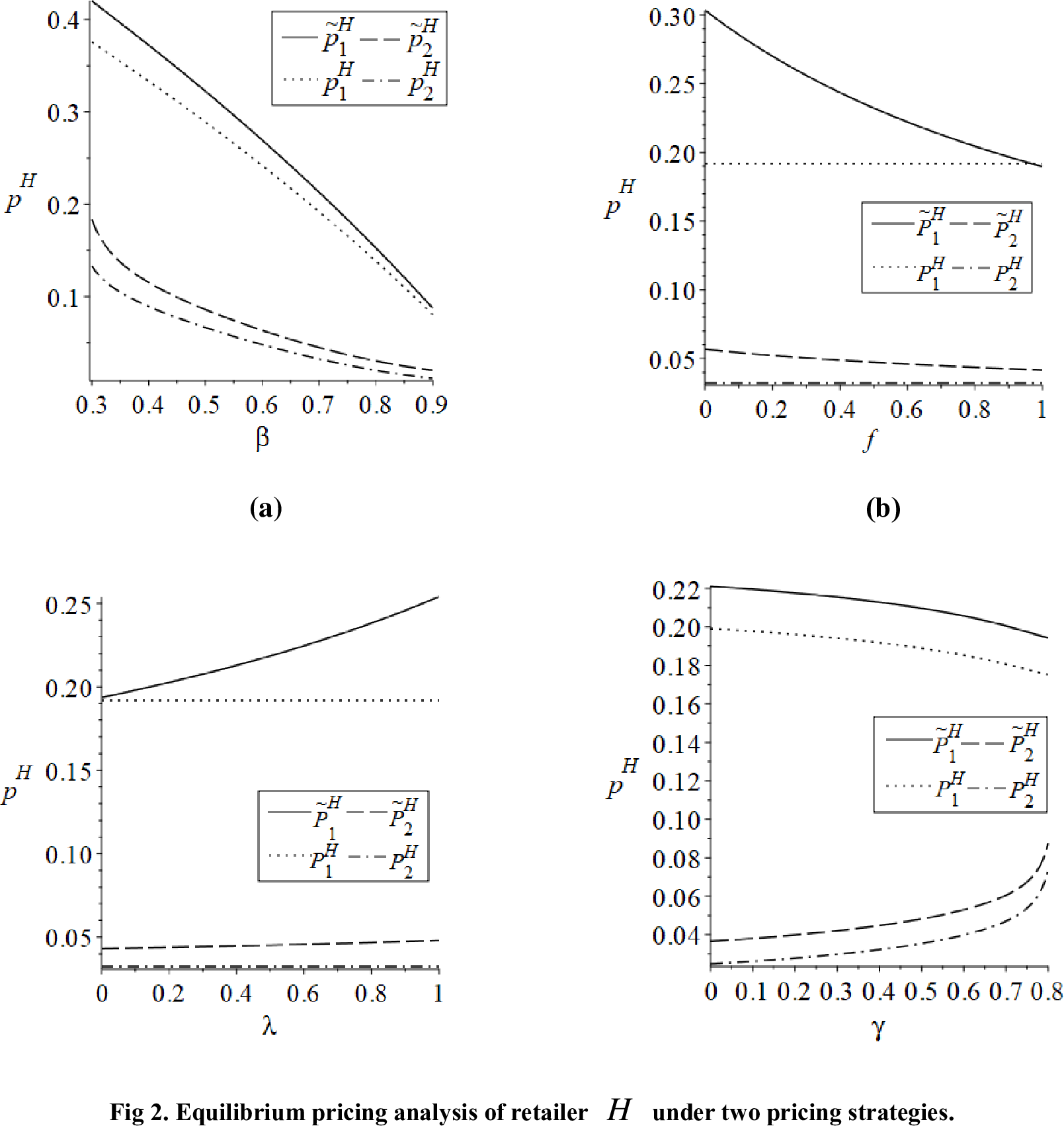

Supplement: S2 Fig — (TIF) [file pone.0296654.s002.tif]

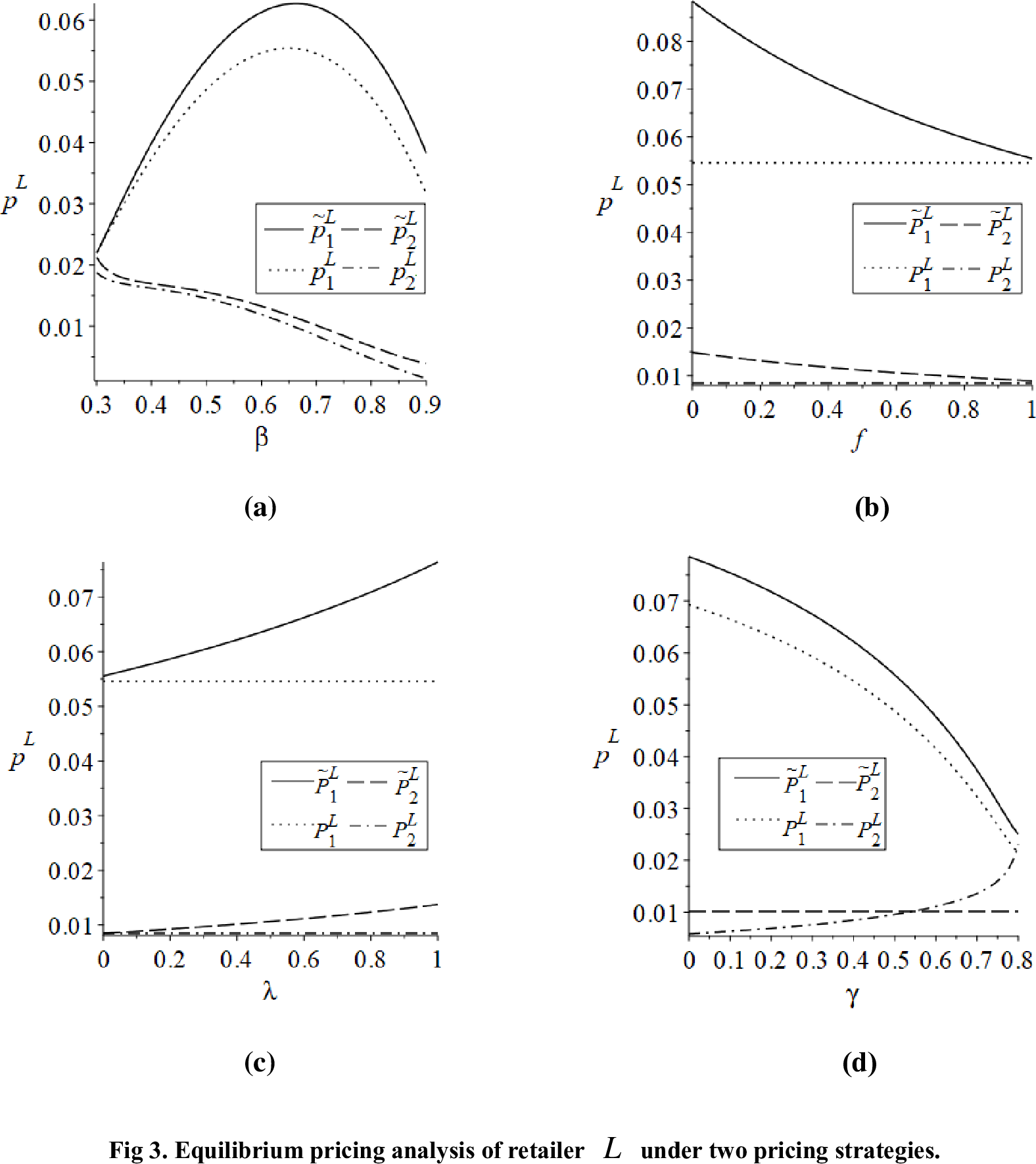

Supplement: S3 Fig — (TIF) [file pone.0296654.s003.tif]

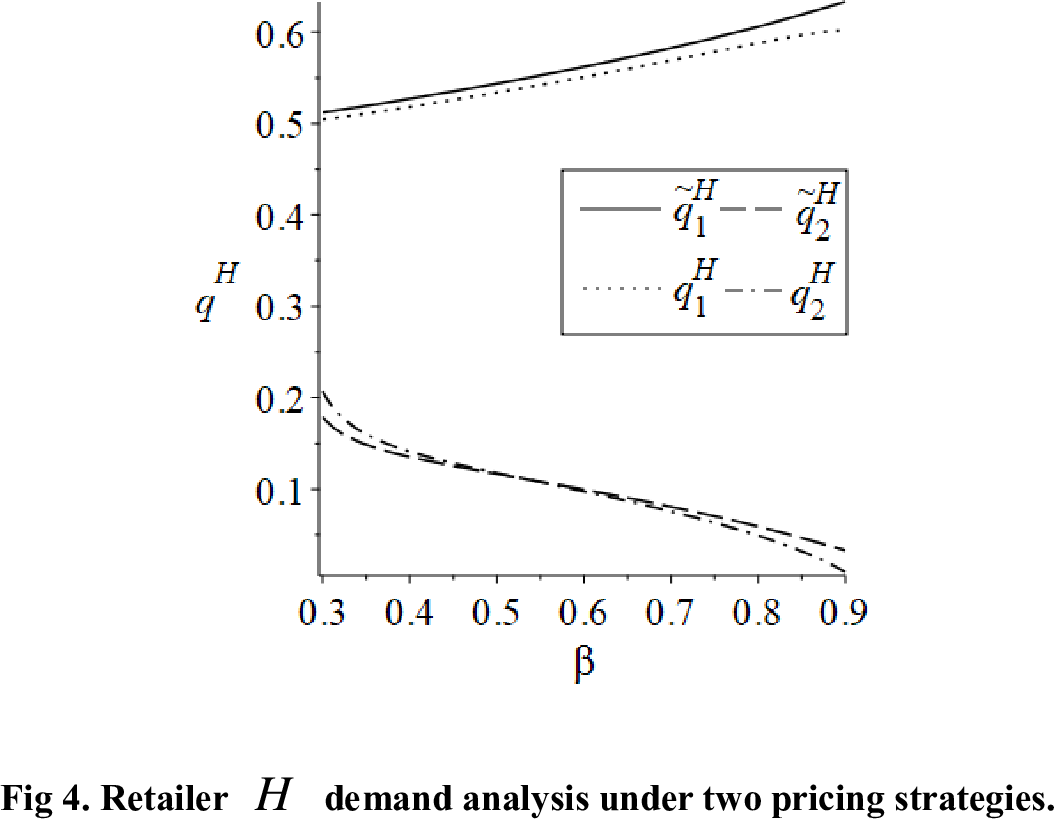

Supplement: S4 Fig — (TIF) [file pone.0296654.s004.tif]

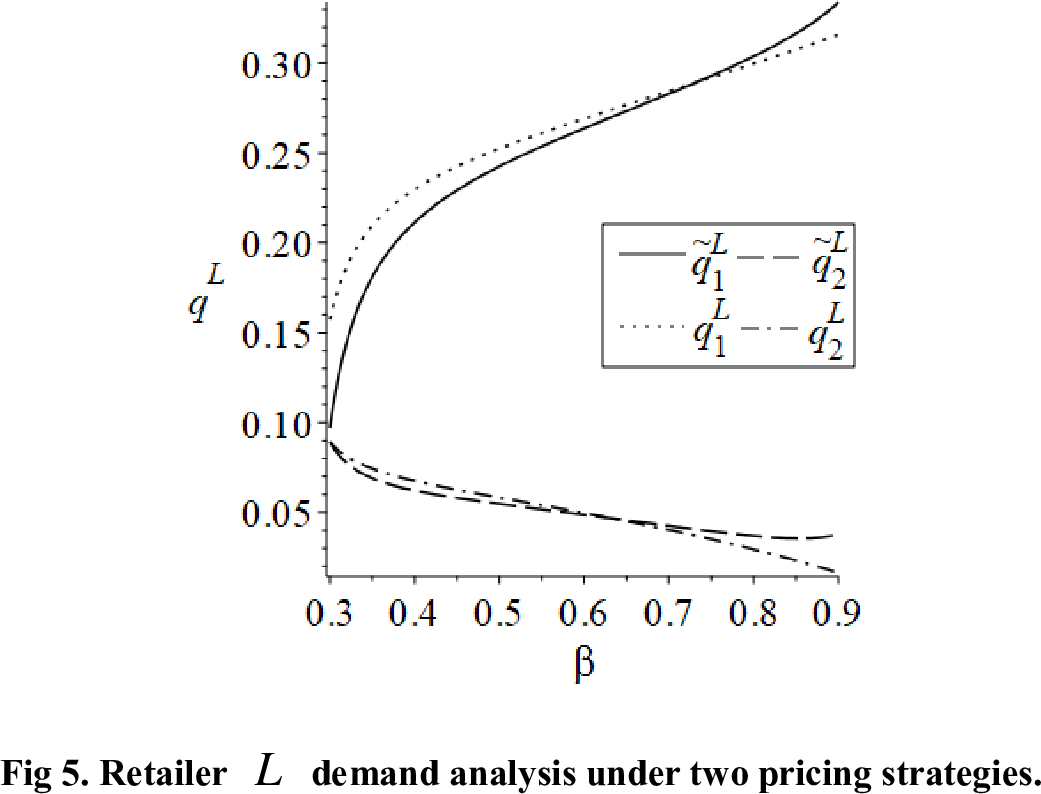

Supplement: S5 Fig — (TIF) [file pone.0296654.s005.tif]

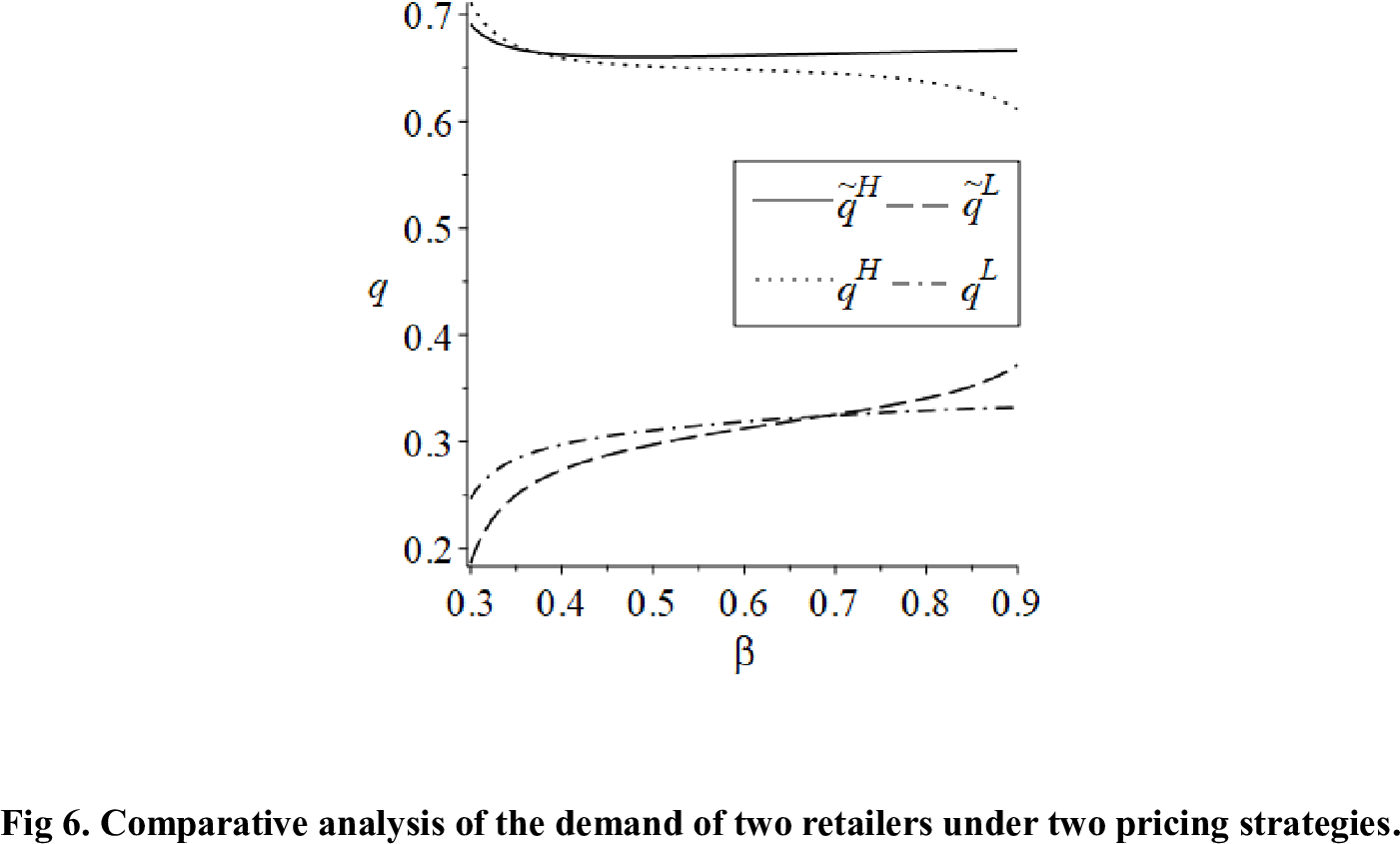

Supplement: S6 Fig — (TIF) [file pone.0296654.s006.tif]

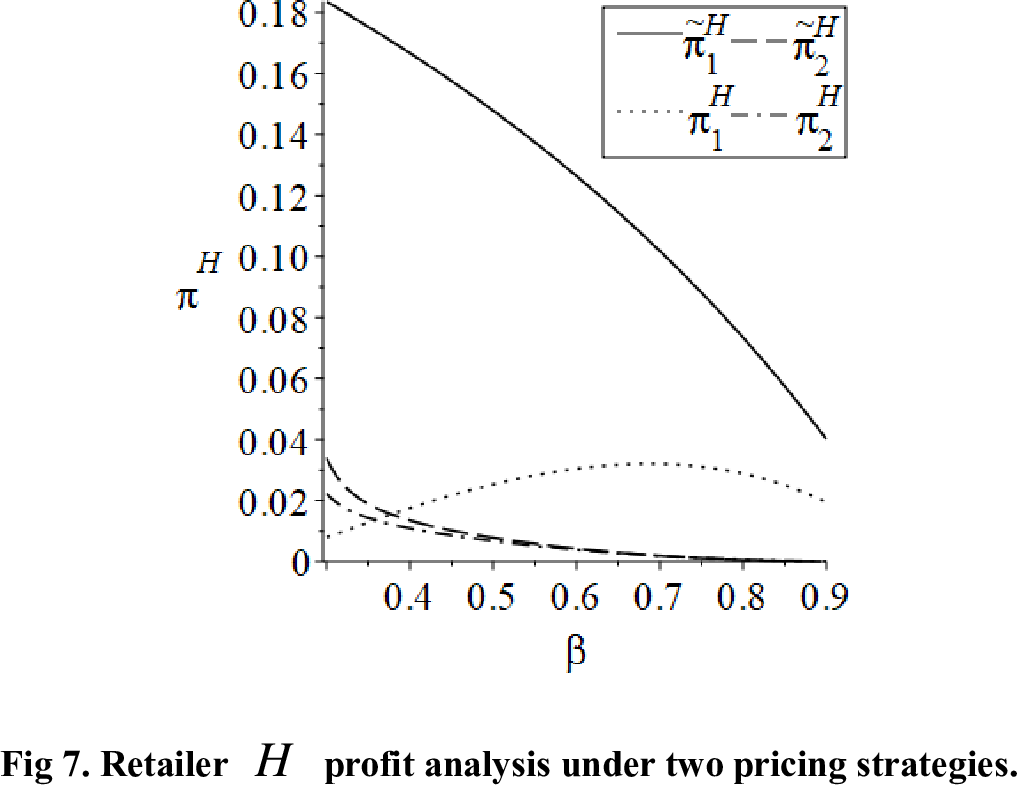

Supplement: S7 Fig — (TIF) [file pone.0296654.s007.tif]

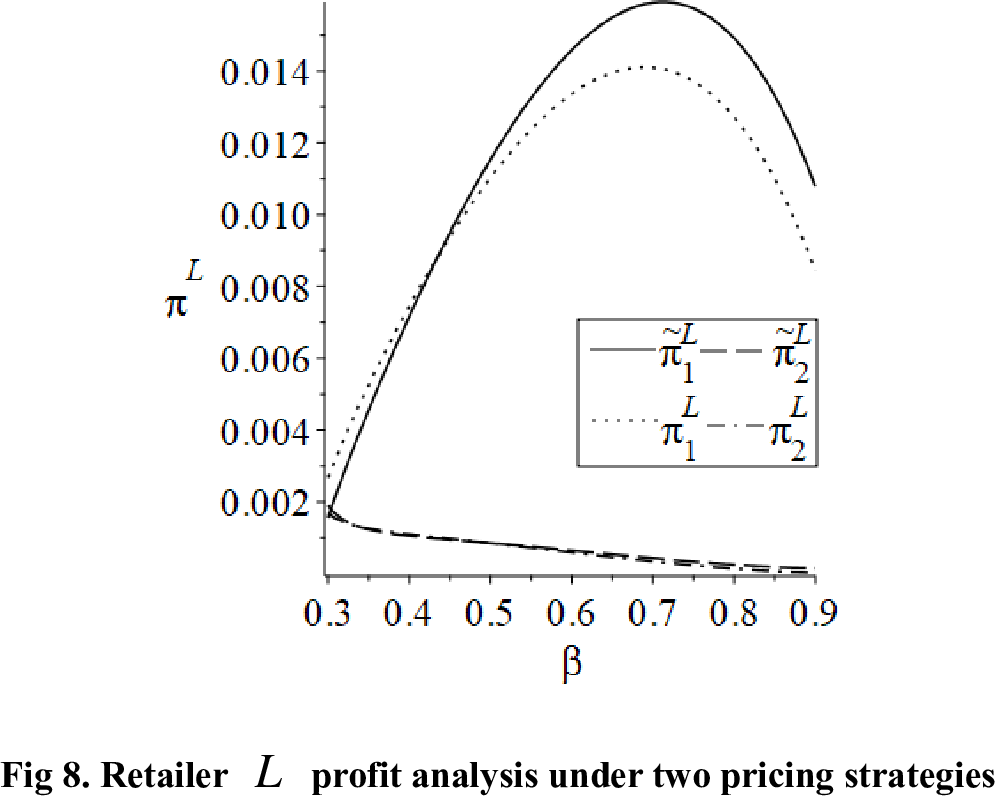

Supplement: S8 Fig — (TIF) [file pone.0296654.s008.tif]

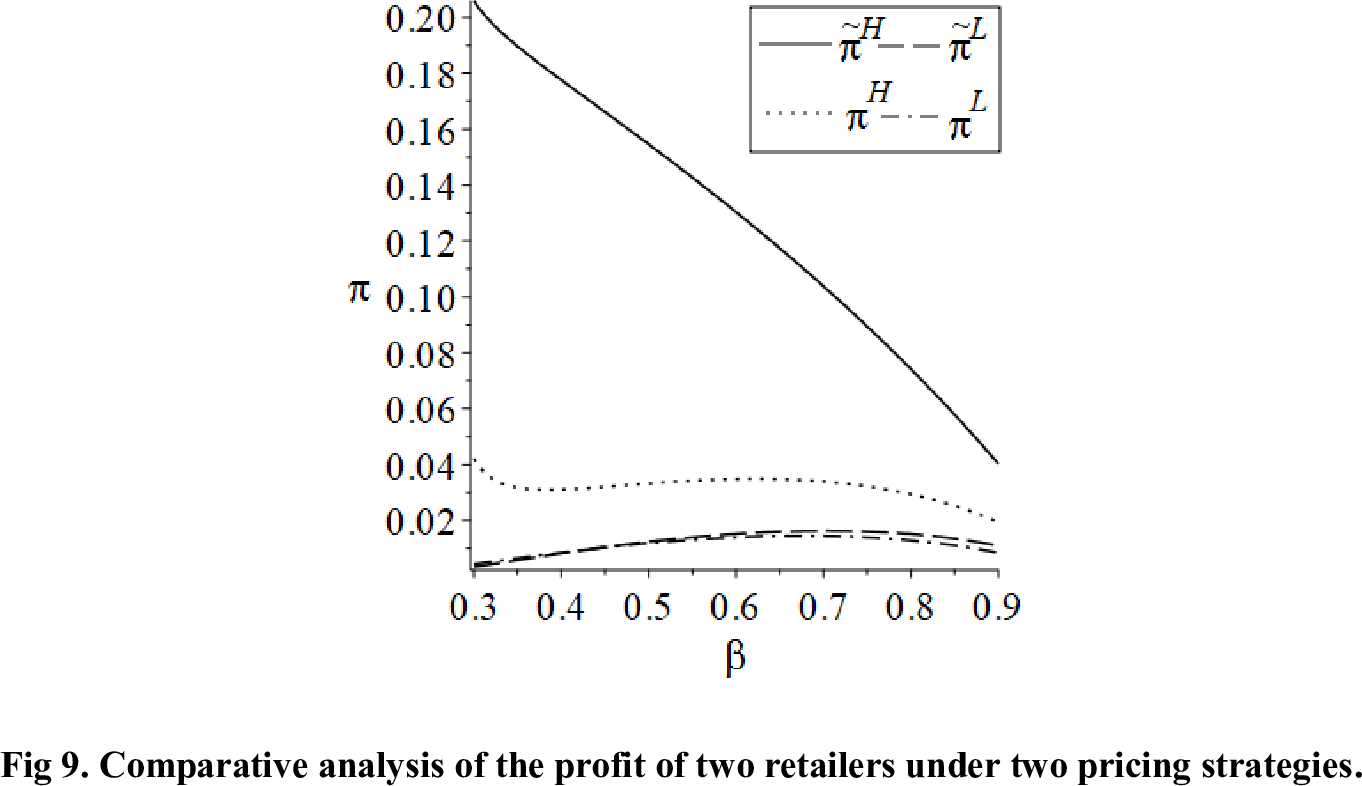

Supplement: S9 Fig — (TIF) [file pone.0296654.s009.tif]

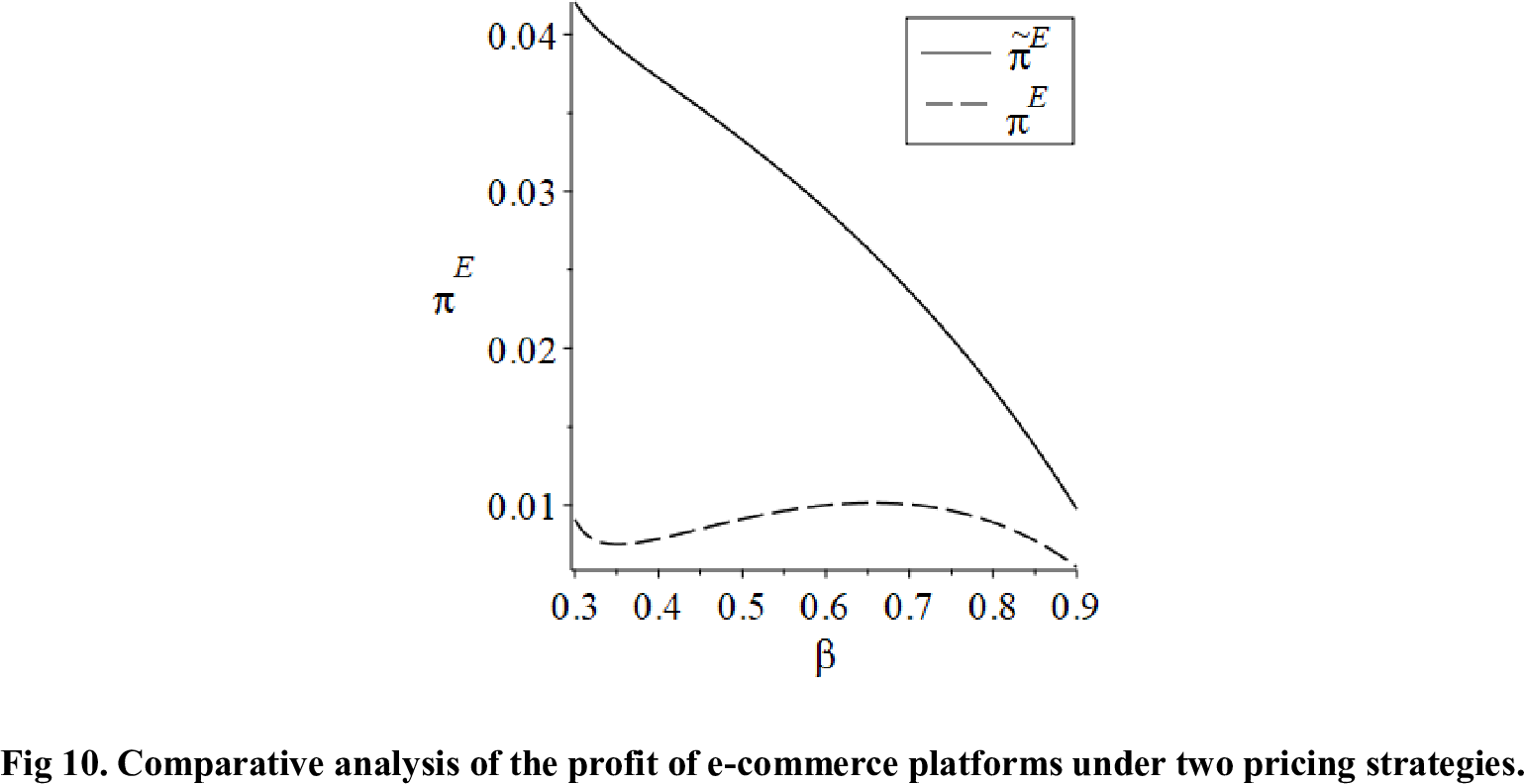

Supplement: S10 Fig — (TIF) [file pone.0296654.s010.tif]

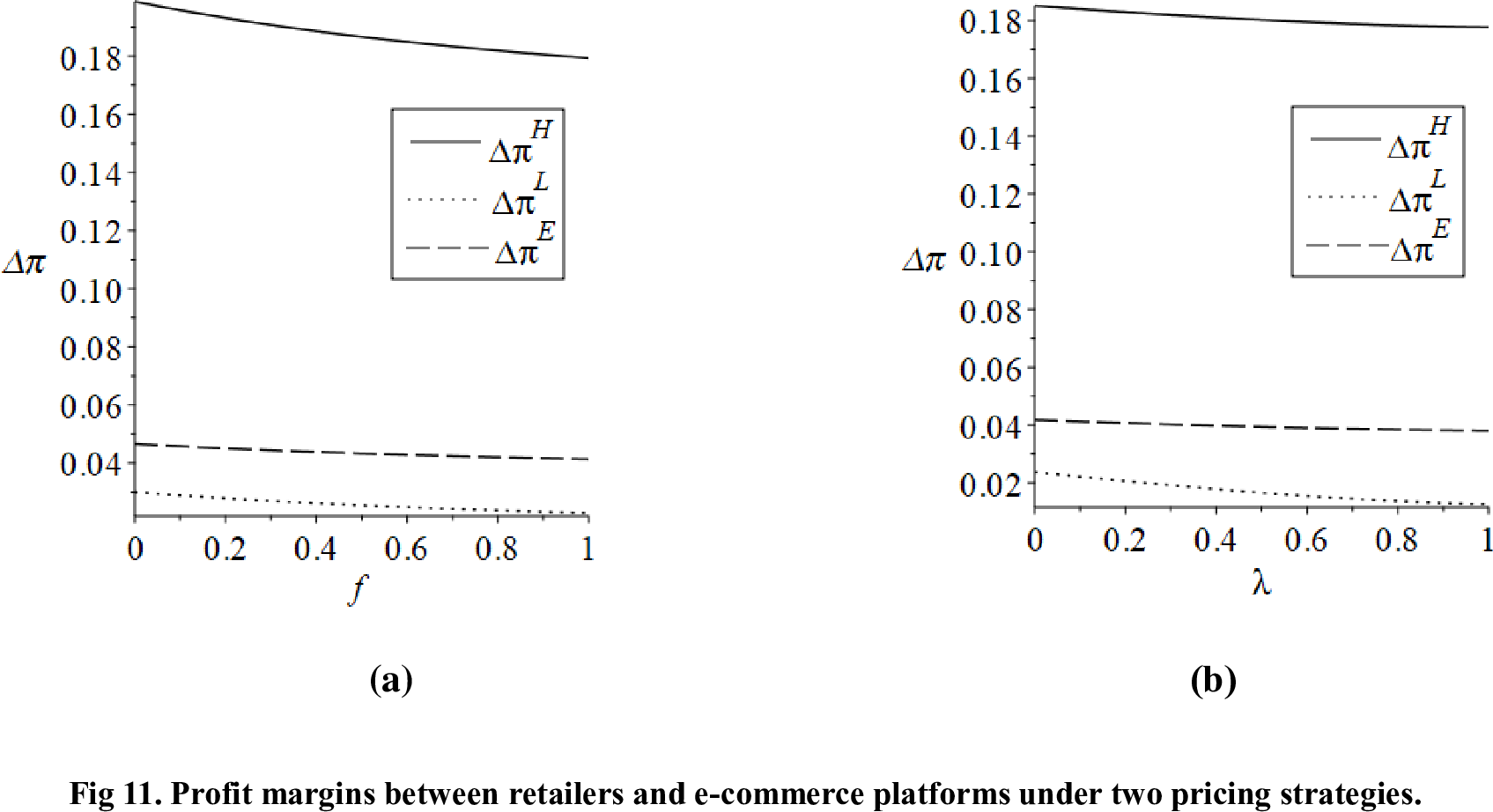

Supplement: S11 Fig — (TIF) [file pone.0296654.s011.tif]
