# Supplementary material for: How do e-commerce platforms and retailers implement discount pricing policies under consumers are strategic?
Source: PLoS One. 2024 May 10;19(5):e0296654. doi: 10.1371/journal.pone.0296654 (PMC11086857; doi:10.1371/journal.pone.0296654)
Supplement: S1 Appendix — (DOCX) [file pone.0296654.s012.docx]

**Appendix**

**Proof of Proposition 1.**

We first solve the first-order condition in the price for Equation (1) and Equation (2) and obtain the following solutions for retailer $H$ and retailer $L$, respectively. The subgame Nash equilibrium of Period 2 can be obtained by satisfying the second partial derivatives less than zero:

$\frac{\partial\pi_{2}^{H}}{\partial p_{2}^{H}}=v_{T}-\frac{2p_{2}^{H}-p_{2}^{L}}{1-\beta}$, $\frac{\partial\pi_{2}^{L}}{\partial p_{2}^{L}}=\frac{p_{2}^{H}-2p_{2}^{L}}{1-\beta}-\frac{2p_{2}^{L}}{\beta}$

The equilibrium price of the two retailers can be obtained jointly. When $0<\beta<1$, the second partial derivatives of $\pi_{2}^{H}$ with respect to $p_{2}^{H}$ and $\pi_{2}^{L}$ with respect to $p_{2}^{L}$ are $-\frac{2}{1-\beta}$ and $\frac{-2}{1-\beta}-\frac{2}{\beta}$. When consumers buy only at retailer $H$, the corresponding profit of retailer $H$ is $\frac{\left( 1-\beta\right)v_{T}}{4-\beta}$ $\left( v_{T}-\frac{\left( 1-\beta\right)v_{T}}{4-\beta} \right)<\pi_{2}^{H*}$. When consumers buy only at retailer $L$, the corresponding profit of retailer $L$ is $\frac{\left( 1-\beta\right)v_{T}}{4-\beta}\left( v_{T}-\frac{\left( 1-\beta\right)v_{T}}{\beta(4-\beta)} \right)<\pi_{2}^{L*}$. We show that neither retailer $H$ nor retailer $L$ have an incentive to deviate. Therefore, Nash equilibrium exists when both retailers have demand.

**Proof of Proposition 2**

With the profit function of both retailers in Equation (3) and Equation (4), solve for the partial derivatives of $p_{1}^{H}$ and $p_{1}^{L}$, respectively. Let $\frac{\partial\pi_{1}^{H*}}{\partial p_{1}^{H}}=0$ and solve for $p_{1}^{H}=\frac{p_{1}^{L}+1-\beta}{2}$, let $\frac{\partial\pi_{1}^{L*}}{\partial p_{1}^{L}}=0$ and substitute into $p_{1}^{H}$, the equilibrium prices of retailers $H$ and $L$ are shown in Equations (5) and (6). It can be judged that the second partial derivative is less than zero, and demand exists when both retailers are sold. Now we show that there exists a Nash equilibrium, and neither retailers has an incentive to deviate from this solution because neither retailer $H$ nor retailer $L$ can deviate to improve profits.

**Proof of Proposition 3**

With the profit function of both retailers in Equation (7) and Equation (8), solve for the partial derivatives of $\tilde{p}_{2}^{H}$ and $\tilde{p}_{2}^{L}$, respectively. Let $\frac{\partial\tilde{\pi}_{2}^{H}}{\partial\tilde{p}_{2}^{H}}=0$ and solve for $\tilde{p}_{2}^{H}=\frac{\left( 1-\beta\right)\tilde{v}_{T}+\left( 1-\lambda+f\lambda\right)\tilde{p}_{2}^{L}-(c_{1}-c_{2})\lambda}{2(1-\lambda+f\lambda)}$, let $\frac{\partial\tilde{\pi}_{2}^{L}}{\partial\tilde{p}_{2}^{L}}=0$ and substitute into $\tilde{p}_{2}^{H}$. The equilibrium price and profit of the two retailers under the double discount are solved as shown in Proposition 3. Calculating the second-order partial derivatives of the retailers' expected profit with respect to the price, we can obtain that both are less than zero. The two retailers' equilibrium profit suffers loss if only one retailer has demand, thus retailers $H$ and $L$ have no incentive to deviate.

**Proof of Proposition 4**

As in the proof of Proposition 1, the partial derivatives of Equation (9) and (10) with respect to $\tilde{p}_{1}^{H}$ and $\tilde{p}_{1}^{L}$ are as follows. Let $\frac{\partial\tilde{\pi}_{1}^{H}}{\partial\tilde{p}_{1}^{H}}=0$ and solve for $\tilde{p}_{1}^{H}$, let $\frac{\partial\tilde{\pi}_{1}^{L}}{\partial\tilde{p}_{1}^{L}}=0$ and substitute into $\tilde{p}_{1}^{H}$. The equilibrium price and profit of the two retailers under a double discount can be obtained as shown in Proposition 4. Calculating the second-order partial derivatives of the retailers' expected profit with respect to the price, we can obtain that both are less than zero. Similar to the proof of Proposition 3, the equilibrium decision of two retailers exists when both retailers sell. The proof process is omitted due to space limitations.
