# Supplementary material for: How do e-commerce platforms and retailers implement discount pricing policies under consumers are strategic?
Source: PLoS One. 2024 May 10;19(5):e0296654. doi: 10.1371/journal.pone.0296654 (PMC11086857; doi:10.1371/journal.pone.0296654)
Supplement: Attachment — Submitted filename: renamed_b9f66.docx [file pone.0296654.s013.docx]

**Review Comments on Manuscript Number:** PONE-D-23-21142

**Title：**How Do E-commerce Platforms and Retailers Implement Discount Pricing Policies under Consumers are Strategic?

**Suggestion: major revision**

This paper constructs a dynamic game model for an e-commerce platform and two retailers when they implement discount pricing, investigate the market equilibrium of the two retailers and e-commerce platform under multiple situations considering consumers’ strategic waiting behavior and competition between the two retailers, and further discuss the effectiveness of retailer discount pricing and the double discount pricing of the platform and retailers. The main results show that the optimal pricing decreases as the difference in product quality narrows under both pricing strategies.

This paper has contributed to the research field of e-commerce supply chain. However, this paper has serious drawbacks, stated as following. Detailed comments are provided below:

- The model description of retailer discount pricing strategy (strategy E) is not very clear. I do not understand the retailer discount pricing. Can you demonstrate which parameter denote the discount pricing? Parameter $\beta$ is the difference quality between the high and low products. So, what’s difference between the period 1 and period 2?
- What’s meaning of $\nu_{T}$ in Eq. (1)?
- In page 20, why do you assume the parameter settings, i.e., $\gamma=0.4$, $f=0.7,\lambda=0.4,c_{1}=0.05,c_{2}=0.03,\beta=0.5,\delta=0.2$? If you change the values of them, whether the main results will be changed?
- The references should follow the format of the Journal.
- For S2 Fig (b) and (c), why the $p_{2}^{H}$ is near 0? Please give some reasons.
- The English level should be improved.
