# Supplementary material for: How do e-commerce platforms and retailers implement discount pricing policies under consumers are strategic?
Source: PLoS One. 2024 May 10;19(5):e0296654. doi: 10.1371/journal.pone.0296654 (PMC11086857; doi:10.1371/journal.pone.0296654)
Supplement: Attachment — Submitted filename: Review comments on PONE-D-23-21142.docx [file pone.0296654.s014.docx]

**How Do E-commerce Platforms and Retailers Implement Discount Pricing Policies under Consumers are Strategic?**

In the era of e-commerce evolution, devising sensible discount strategies for e-commerce platforms and retailers is crucial for maintaining a sustainable business. Drawing on the literature related to consumer strategic behavior and discount pricing strategies, this paper constructs a dynamic game model for the e-commerce platform and two retailers. The model investigates market equilibrium under various scenarios, factoring in consumers’ strategic waiting behavior and the impact of retailer discount pricing and dual discounts from the platform and retailers. This is an urgent challenge faced by retailers and e-commerce platforms. Although the author has made substantial efforts, from a professional standpoint, I believe this paper does not yet meet the publication standards of the PLOS ONE journal. Therefore, I recommend a "**Reject**" decision and provide specific review comments as follows:

1. The paper lacks sufficient innovation and some of the theoretical contributions put forth by the authors do not hold up. To elaborate:

(1) Mu et al. (2020) already investigated the optimal pricing strategy for merchants and e-commerce platforms, taking into account consumer rebates and the dual discount phenomenon. Liu and Zhang (2013) analyzed a market with two firms, *H* and *L*, both offering quality-differentiated products with $q_{H}$ and $q_{L}$ respectively.

(2) Furthermore, in your discussion of Strategy E, it's unclear how the retailer's offer of a discount is factored in. What is the precise relationship between $p_{1}^{i}$ and $p_{2}^{i}$? Is it correct to infer that your paper strictly contemplates whether or not the platform provides rebates?

[1] Mu L, Wang F, Chen L. Research on pricing strategy of E-commerce platform based on strategic consumers[J]. Operations Research and Management Science, 2020, 29(10): 225.

[2] Liu Q, Zhang D. Dynamic pricing competition with strategic customers under vertical product differentiation[J]. Management Science, 2013, 59(1): 84-101.

2. There seem to be issues with your model, with certain expressions appearing ambiguous and confusing.

(1) The range of some parameters needs additional scrutiny. Specifically, "($q_{H}$, $q_{L}$) to (1, *β*) with $\beta\in\left[ 0, 1 \right]$", where *β* cannot reach the value of 1 as $q_{H}$>$q_{L}$, and cannot be 0 either, since β might serve as the denominator.

(2) In Strategy E, could you provide further proof to validate that $v_{2}^{L}$ fulfills the condition $v_{2}^{L}=\frac{p_{2}^{L}}{\beta}$?

(3) The statement "defining the rebate rate of the e-commerce platform as *f*, consumers can enjoy a discount of (1-*f*)" is perplexing. The definition of the rebate rate and discount seem rather similar. For instance, if a product is priced at ￥100 and has a rebate rate of "10%", it implies you pay ￥100 and receive ￥10 back, which means you effectively enjoy a discount of 10% and only need to pay ￥90. Consequently, consumers can enjoy a discount of "*f*", resulting in the actual price paid, (1-*f*)$\tilde{p_{2}^{i}}$, under both platform and retailer discounts. Moreover, typically a discount is deducted directly at the time of purchase, while a rebate is returned to the consumer after purchase at a certain rate, a characteristic that this paper's model fails to represent effectively.

3. The use and citation of references and supporting content in the manuscript is incorrect. The citation marked as [27] in the manuscript states, "We can interpret $\gamma$ as the level of strategic behavior (Prasad et al. [27]), and a higher γ means……". However, the actual reference is not [27] but [13], which states "Hence, $\gamma$ can be interpreted as the level of customer’s strategicity/rationality; a higher $\gamma$ implies that customers are more strategic" (Liu and Zhang 2013). In the manuscript, the authors mention that gamma represents the valuation discount factor for strategic waiting by the parameter, and thus, gamma should only affect value '*v*', not '(*v*-*p*)'? In addition, strategic customer behavior (i.e., $\gamma$) does not play any role in the last period game because there are no future purchase opportunities (Liu and Zhang 2013).

4. The parameter values in the numerical examples lack practical case support, and there should be additional clarification regarding the data sources. The statement that "the optimal pricing of retailer L in the first period decreases before it increases" does not align with Fig. 3(a). The conclusions drawn from the research are quite intuitive, lacking in intriguing findings and valuable managerial insights.

5. Several issues related to English phrasing, grammar, and input errors require a comprehensive review and rectification throughout the text to prevent inaccuracies and increase readability. Therefore, it is recommended that the article undergo a thorough proofreading process. Illustrations of the points that need attention are as follows:

(1) The precision of the language needs to be enhanced. In the "Abstract" section, there are grammatical errors, for example, " In this paper, we constructs a dynamic game……，investigate the……，and further discuss the……" Also, in the "Numerical Study and Managerial Insights" section, the sentence, "The second factor is (the) rebate (rate), which is summarized by the parameter f," needs correction.

(2) Furthermore, some English expressions are challenging to understand. The phrase "Apply for refunding the deposit for presale product after receiving the goods" may not be a suitable example to illustrate the concept of rebate redemption in today's context, as the deposit is typically part of the final payment in reality. Sentences such as "An increase in the rebate rate means that consumers receive fewer discounts from the platform and the retailer will choose to reduce prices to attract consumers " and " The greater the rebate rate is, the smaller the discount on the platform." require refinement.
